# Linking Out Loud (LOL): Developing critical thinking

**DOI:** 10.15694/mep.2020.000042.1

**Published:** 2020-03-10

**Authors:** Wendy Foster, Linda Sweet, Liz McNeill

**Affiliations:** 1 University of South Australia; 2 Deakin University; 3 Flinders University

**Keywords:** critical thinking, gamification, midwifery, learning

## Abstract

This article was migrated. The article was marked as recommended.

**Background:** Developing critical thinking in health care education is pivotal in student development and patient outcomes. Gamification and learning through play within health care education is rising in popularity and may assist to develop problem-solving and critical thinking skills.

**Aim:** To develop and enhance students’ capacity to rapidly recall and communicate critical links in practice through a modified ‘snap’ game.

**Method:** Students are provided decks of cards depicting clinical pictures (syringe, bath, blood, medications etc). Turning over two cards at a time, the students race to link the two concepts together.

**Findings:** Linking Out Loud was well received by students and worked to develop knowledge, critical thinking, communication and problem-solving. Linking Out Loud could be easily replicated for all health disciplines.

## Background

Critical thinking is the process of making objective analysis and evaluation of an issue. Critical thinking requires high order cognitive processing that facilitates contextual decision-making and problem-solving and is critical for all health care practice. Developing critical thinking has been identified as assisting to improve patient outcomes and increasing levels of job satisfaction ( Alfaro-LeFevre, 2016). Higher education organisations tout critical thinking as being one of their graduate qualities; however, the development of critical thinking is inherently difficult. Despite the clear benefits of critical thinking, it has been identified as difficult to teach, requiring deep level thinking and can be impacted on by the knowledge base level specific to the subject area ( Willingham, 2008).

Gamification is a relatively new concept in health professional education ( Brull and Finlayson, 2016), however, it is rapidly becoming recognised for its potential to improve student learning ( Castro and Gonçalves, 2018). Known benefits include autonomous learning, non-threatening and safe learning environments, development of problem-solving skills, and increased knowledge retention rates ( Castro and Gonçalves, 2018, Woolwine, Romp and Jackson 2019). Using gamification theory, we developed a ‘Linking-out-Loud’ game for midwifery students. It encourages students to draw on their own clinical and theoretical experiences and understandings to spark clinical debate and discussion, and develop critical thinking skills.

## Aim

The aim of this project was to develop students’ capacity for rapid recall and critical thinking, and identify factors influencing midwifery care through gamification. A secondary aim was to develop and enhance the students’ ability to communicate effectively while sharing knowledge and understanding, while linking theory with practice.

## Method

The ‘Linking-out-Loud’ game is based on the card game ‘snap’. In this game, students work in small teams of up to five. Each team is provided with a deck of approximately 50 cards, each containing a different image depicting a clinical midwifery situation, product or concept. Images of items such as a medication, a catheter, bed, bath or a picture of a pregnant woman are used. The students place the shuffled cards face down in a deck. The first student turns one card and the second student turns another. With two cards visible, the two students look at the images displayed and then race to ‘snap’ on the cards once they can provide a link between them. This creates anticipation, relying on rapid recall and provides low-stress competitive fun. It is important that the two students flipping the cards are the first to identify and explain a link. Following this, the other members of the team in observer roles, may question the link, provide a different link, or expand on the initial link. Students are encouraged to discuss their own point of view and provide rationales while respecting the opinions and experiences of other students. Students rotate to be the person who is turning a card and thus getting the opportunity to do the ‘Linking out Loud’ first.

As an example, two cards are placed face-up on the table. One is of a baby being breastfed, while the other image is oxytocin. One student’s response for this combination might be ‘ *the use of oxytocin for induction of labour may impact on lactogenesis, meaning additional breastfeeding support and hand expressing may be required’.* Another student may expand on this point by adding ‘ *This is due to the synthetic oxytocin inhibiting the synthesis of endogenous oxytocin’.* While another student may link the two statements together such as, ‘ *Oxytocin is responsible for the letdown reflex and milk ejection. This is due to the contractile nature of oxytocin working on the milk ducts within the breast*’. In our experience, in subsequent classes on birthing complications in the following semester, one student commented on the risk between an induction of labour with oxytocin and the risk of postpartum haemorrhage. She followed this by describing the influence of the postpartum haemorrhage on breastfeeding. In this example, the sharing of knowledge and ideas strengthened the groups’ understanding of the physiological influence of oxytocin. This highlights the sharing of ideas as well as rapidly deducing care considerations from the two concepts. A student in second year may have a focus on breastfeeding establishment and the normal physiological process, while a third-year student may be able to deduce complex connections such as the latter response.

## Findings

The ‘Linking-out-Loud’, nicknamed ‘LOL’ game has received positive evaluations from both second- and third-year students to date. Students appreciated the dynamic nature of the game and the sharing of ideas. They enjoyed the teamwork and found it interesting to hear how others would link concepts. Whilst established with midwifery student, this game can be modified to all health professions, and may be used as an icebreaking activity for interprofessional learning as this may encourage sharing of professional values and understanding. Linking-out-Loud is an easily adaptable, flexible and enjoyable way to develop critical thinking and the development of professional relationships.

## Take Home Messages

• Critical thinking can be difficult to teach
• Gamification processes encourage interaction and create a fun learning space
• The Linking Out Loud game promotes declaration of knowledge and rationales
• The Linking Out Loud game promotes co-construction of knowledge
• The Linking Out Loud card deck can be used across all levels of learners

## Notes On Contributors

Wendy Foster: BMid, Grad Cert Clin.Ed, MMid, PhD Candidate, Midwifery Lecturer.

Linda Sweet: BN, MNgS, PhD, Grad Cert Education (Higher Ed). Chair in Midwifery Deakin University and Western Health Partnership. ORCiD: https://orcid.org/0000-0003-0605-1186

Liz McNeill: RN/RM BHSc(Nurs), BMid, GCEd(Tert Ed), GCCritCareNurs, GC FlexEd&Simulation, MNg, PhD Candidate. Midwifery Lecturer. ORCiD: https://orcid.org/0000-0003-3640-6494
