# Supplementary material for: Linking Out Loud (LOL): Developing critical thinking
Source: MedEdPublish (2016). 2020 Mar 10;9:42. [Version 1] doi: 10.15694/mep.2020.000042.1 (PMC10697476; doi:10.15694/mep.2020.000042.1)
Supplement: Peer review history

# Reviewer report: Reviewer response for version 1

**DOI:** 10.21956/mep.20005.r30490

**Published:** 2021-04-02

**Authors:** Davinder Sandhu

**Affiliations:** 1 Royal College of Surgeons in Ireland, Medical University of Bahrain, POBox 15503, Adliya, Bahrain.

This review has been migrated. The reviewer awarded 3 stars out of 5 The authors are to be congratulated on bringing in the concept of gamification in developing higher order cognitive function of critical thinking. The authors could have expanded more on educational theory directly linking critical thinking to clinical practice. Learning is the critical aspect and is a new way of doing things.Learning is not something done to students, but rather something students do for themselves. It is the direct result of how students interpret and respond to their experiences. Learning is enhanced by two critical elements of motivation and reflection which comes from self-determination theory. Clinical practice depends upon clinical reasoning which is the thinking and decision making processes. This leads to clinical thinking where knowledge; skills; observation and judgement informs patient management. The final path is to critical thinking which involves the analysis of facts to form a judgement, the ability to interpret argument, evidence or raw information in a logical and unbiased fashion.In other words the ability to solve complex problems effectively. That is where I feel the strength of gamification lies in problem solving.

Reviewer Expertise:

NA

No decision status is available

# Reviewer report: Reviewer response for version 1

**DOI:** 10.21956/mep.20005.r30493

**Published:** 2020-04-03

**Authors:** Shaoting Feng

**Affiliations:** 1 The First Affiliated Hospital of Sun Yat-sen University

This review has been migrated. The reviewer awarded 3 stars out of 5 This study shows us a novel approach to teaching critical thinking. I believe that the ‘LOL’ game can encourage interaction and create a fun learning space for students. However, it seems that the effectiveness of developing critical thinking though this ‘LOL’ game should be further studied and discussed.

Reviewer Expertise:

NA

No decision status is available

# Reviewer report: Reviewer response for version 1

**DOI:** 10.21956/mep.20005.r30489

**Published:** 2020-03-22

**Authors:** P Ravi Shankar

**Affiliations:** 1 American International Medical University

This review has been migrated. The reviewer awarded 4 stars out of 5 This is an interesting study. The authors have described the use of an interesting card game using the technic of Linking Out Loud (LOL). They have described the game and the process of playing it in detail. We had used a card game ‘Braincept’ among medical students and initial results showed an improvement in knowledge retention and clinical application. The authors mention that student feedback about the game was positive. They also mention that participants enjoyed the group interaction facilitated by the game. This is a preliminary evaluation and long-term studies may be required. I would be interested in knowing initial participant feedback about the game. As an additional resource the authors can add a video on how to play the game. I am not sure whether MedEdPublish in the current format can support videos but the authors can definitely add a hyperlink. The article will be of interest to a broad section of educators as with modifications the game can be used among medical, nursing and other health science students.

Reviewer Expertise:

NA

No decision status is available

# Reviewer report: Reviewer response for version 1

**DOI:** 10.21956/mep.20005.r30492

**Published:** 2020-03-16

**Authors:** Susan Van Schalkwyk

**Affiliations:** 1 Stellenbosch University

This review has been migrated. The reviewer awarded 3 stars out of 5 The notion of employing elements of play in the learning context (gamification) offers alternative opportunities for facilitating learning. It is important that we engage thoughtfully with what these affordances bring and explore how they influence student learning. This article focusses on a gamification intervention adopted to promote ‘critical thinking’. An argument is made for the importance of critical thinking in the higher education context, while acknowledging that teaching such thinking can be difficult. In response, a game has been developed which is described as ‘an easily, adaptable, flexible and enjoyable way to develop critical thinking …’. While the game that has been designed does indeed sound innovative, and I can imagine that students would enjoy it, I do have further questions and suggestions for further investigation. For example, it would have been helpful to have been provided with a bit more information as to how the game plays out in practice. How many groups of five would there typically be in any particular session? Is the lecturer/teacher/tutor available to observe the process/provide guidance if the discussion goes off track? What happens if students are unable to identify the link? There is no doubt that sharing of ideas, hearing how others see linkages, and having to essentially justify one’s own thinking, ought to have value for student learning. I would, however, agree with earlier reviewers that further, follow up work would be important to strengthen the claims being made. As an aside, I specifically liked the idea of using something like this as an ice-breaker during interprofessional learning activities.

Reviewer Expertise:

NA

No decision status is available

# Reviewer report: Reviewer response for version 1

**DOI:** 10.21956/mep.20005.r30491

**Published:** 2020-03-12

**Authors:** Puja Dulloo

**Affiliations:** 1 Pramukhswami Medical College

This review has been migrated. The reviewer awarded 3 stars out of 5 The topic is appropriate for medical education explaining the newer approach for developing a critical thinking approach in health science students. The abstract is accurate and well described in a concise manner.The introduction part is too compressed. More elaboration for the gamification theory should have been in the background with the justification of using this theory over and about other techniques for the group of participants.The methodology was well explained except how it was made sure that the two cards had a sort of connection or link. Maybe the two cards opened were not connected, so in that case what instructions were given to students. The focus should have been on the monitoring of the students; if they were linking it correctly. What if they got stuck at any point in time? It was highlighted in the aim that participants were students undertaking midwifery care program but the level or milestone of the participants was not highlighted. Where 1st, 2nd and 3rd-year students in the same group or different.The result of the study has not been represented. The finding of the study seems to be the conclusion of the study. I would love to know about what percentage of students' appreciated the dynamic nature of the game prepared and identify the linking and sharing of information as per the displayed cards.

Reviewer Expertise:

NA

No decision status is available

# Reviewer report: Reviewer response for version 1

**DOI:** 10.21956/mep.20005.r30488

**Published:** 2020-03-11

**Authors:** Trevor Gibbs

**Affiliations:** 1 AMEE

This review has been migrated. The reviewer awarded 3 stars out of 5 Although not a great fan of gamification, I did enjoy reading this paper and appreciated its novelty and its approach to active learning. My worry however , and this applies to this paper, is how much real value and effect we place on activities like this. I am sure that the students enjoyed the game and I am sure that somewhere in there , there was an increase in knowledge and possible clinical thinking. I do believe however that the real educational value can only be judged by evaluating the effect upon the students applied learning- does this apparent new learning and exploration lead to change ( learning is about changing behaviour) and is this / can this change be applied to real situations. I think that this is only the start of this piece of research and a follow up of students into their clinical years and later would add greater value to this work.

Reviewer Expertise:

NA

No decision status is available
